# Tβ4‐Engineered ADSC Extracellular Vesicles Rescue Cell Senescence Through Separable Microneedle Patches for Diabetic Wound Healing

**DOI:** 10.1002/advs.202505009

**Published:** 2025-04-25

**Authors:** Youjun Ding, Jinglin Wang, Jiaye Li, Yi Cheng, Shuyin Zhou, Yepeng Zhang, Yuanjin Zhao, Min Zhou

**Affiliations:** ^1^ Department of Vascular Surgery Cardiovascular medical center Nanjing Drum Tower Hospital Clinical College Jiangsu University Nanjing 210002 China; ^2^ State Key Laboratory of Bioelectronics,School of Biological Science and Medical Engineering Southeast University Nanjing 210096 China; ^3^ Department of Emergency Surgery The Fourth Affiliated Hospital of Jiangsu University (Zhenjiang Fourth People’s Hospital) Zhenjiang 212002 China

**Keywords:** cell senescence, extracellular vesicle, microneedle, stem cell, wound healing

## Abstract

Microneedles loaded with bioactive substances have demonstrated efficacy in wound healing, while their application in the elderly chronic wounds, aggravated by cellular senescence, is still a significant challenge. Here, a novel therapeutic strategy is presented utilizing Thymosin β4 (Tβ4)‐modified adipose‐derived stem cell extracellular vesicles (ADSC‐EVs) delivered via separable microneedle patches (MN@EVs^Tβ4^). The therapeutic EVs^Tβ4^ are derived from ADSCs that overexpress Tβ4, a factor that reverses cellular senescence. Leveraging the drug‐loading and release properties of gelatin methacryloyl and poly(ethylene glycol) diacrylate, EVs^Tβ4^ are encapsulated within the tips of the microneedles. Notably, the soluble hyaluronic acid base layer dissolves rapidly and separates from the tips upon exudate absorption, enabling a sustained release of EVs^Tβ4^. Subsequently, it is demonstrated its ability to mitigate senescence and improve function via the PTEN/PI3K/AKT pathway. Furthermore, MN@EVs^Tβ4^ patches showed significant efficacy in reversing senescence and promoting wound healing in diabetic wound models. Thus, the engineered ADSC‐EVs, combined with separable microneedle patches, represent a promising bioengineering strategy for clinical wound management.

## Graphical abstract

A separable microneedle patch is designed for Tβ4–modified ADSC‐derived extracellular vesicles delivery. The overexpressed Tβ4 released from the microneedles can mitigate cellular senescence and promote wound healing via the PTEN/PI3K/AKT pathway, offering a promising approach for diabetic wound treatment. This bioengineered therapeutic system serve as a promising strategy for clinical wound management.

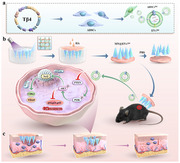

## 1 Introduction

Diabetic wounds represent a prevalent and challenging health issue, particularly among the elderly population.^[^ ^1^ ^]^ Unlike typical wounds, those in older adults often resist conventional healing strategies despite the application of numerous stimulatory substances.^[^ ^2^ ^]^ This resistance is attributed to the advanced cellular senescence, which diminishes the effectiveness of traditional wound healing approaches.^[^ ^3^ ^]^ Microneedle patches, which can be loaded with various factors and bioactive substances, have emerged as a promising treatment modality for diabetic wounds.^[^ ^4^ ^]^ However, while these patches show potential in preclinical models, their efficacy in real‐world scenarios is often constrained.^[^ ^5^ ^]^ The primary limitation stems from the complex interplay of cellular aging and other factors that defy the reparative capabilities of conventional therapies. Consequently, there is a compelling need for innovative therapeutic technologies that can effectively address the intricacies of diabetic wound healing in the elderly.

**Figure 1:**
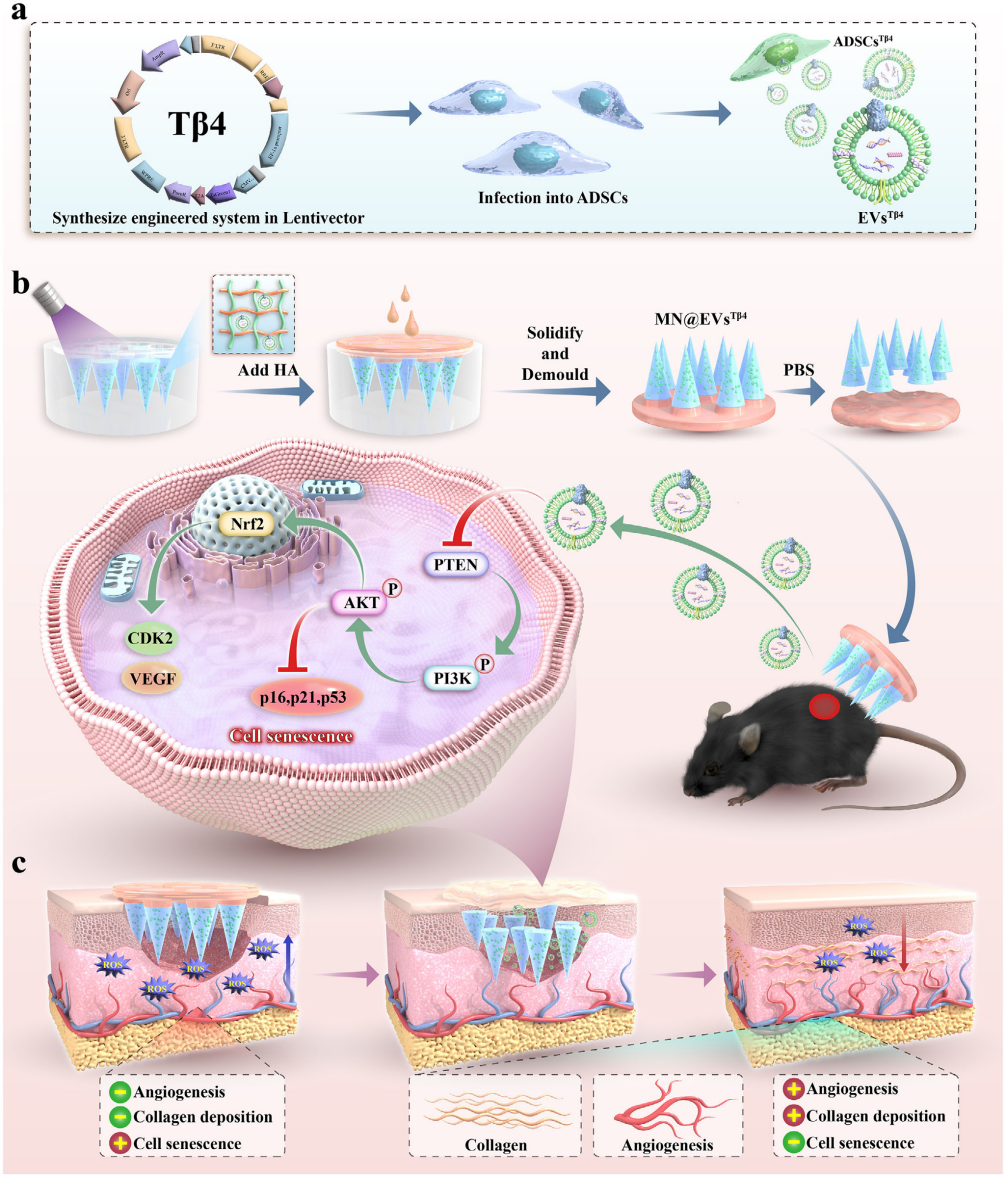
Schematic diagram illustrating the fabrication and application of EVs^Tβ4^‐loaded microneedle patches for facilitating wound treatment. a) EVs^Tβ4^ was prepared via lentivirus‐mediated gene transfection. b) The separable microneedle MN@EVs^Tβ4^ patch was prepared via a two‐step template replication technique. c) The MN@EVs^Tβ4^ patch rescued cell senescence and improved the function of damaged cells through the PTEN/PI3K/AKT signaling pathway, thereby accelerating wound healing.

Here, we introduce a novel therapeutic strategy for diabetic wound healing that leverages Thymosin β4 (Tβ4)‐modified adipose‐derived stem cell extracellular vesicles (ADSC‐EVs) delivered via separable microneedle patches, as schemed in **Figure** **1**. ADSCs, known for their ease of accessibility and potent immunosuppressive properties, are recognized as highly effective cells for the treatment of diabetic wounds.^[^ ^6^ ^]^ Compared to ADSCs, their derived extracellular vesicles (EVs) demonstrate superior malleability and manipulability.^[^ ^7^ ^]^ Notably, EVs can encapsulate bioactive substances, facilitating the delivery to recipient cells and enabling the sustained release of therapeutic cytokines.^[^ ^8^ ^]^ In contrast, Tβ4, as a factor that mitigates cellular senescence, is considered a key player in the treatment of various age‐related diseases.^[^ ^9^ ^]^ High expression of Tβ4 can counteract the aging tendencies of numerous cell types, such as cardiomyocytes and endothelial cells.^[^ ^10^ ^]^ Therefore, we hypothesize that engineering Tβ4 into ADSC‐EVs could develop an effective strategy to reverse cellular aging. Based on this concept, we aim to utilize microneedle delivery to achieve a long‐lasting, sustained release therapy for diabetic wounds.

In this study, we implemented a therapeutic strategy for diabetic wound healing by engineering EVs derived from Tβ4‐overexpressing ADSCs, which were then integrated into microneedle patches (MN@EVs^Tβ4^). Through lentiviral transduction, ADSCs were genetically modified to overexpress Tβ4, yielding EVs^Tβ4^ that were efficiently internalized by recipient cells. By leveraging the drug‐loading and controlled‐release properties of gelatin methacryloyl (GelMA) and poly(ethylene glycol) diacrylate (PEGDA), we engineered microneedle tips to encapsulate EVs^Tβ4^. Notably, the base layer of the microneedles was composed of soluble hyaluronic acid (HA), which dissolved rapidly upon absorption of exudate, allowed the MN@EVs^Tβ4^ tips to continuously and stably release EVs^Tβ4^ within the diabetic wound. Mechanistically, EVs^Tβ4^ alleviated hyperglycemia‐induced cellular senescence by enhancing cellular functionality through activation of the PTEN/PI3K/AKT pathway. In diabetic mouse wound models, MN@EVs^Tβ4^ patches significantly reduced senescence markers, attenuated oxidative stress, promoted angiogenesis, and enhanced collagen deposition. Collectively, our separable microneedle system provides an effective platform for delivering ADSC‐derived EVs, expanding their translational potential in clinical wound management.

## 2 Results and Discussion

**Figure 2:**
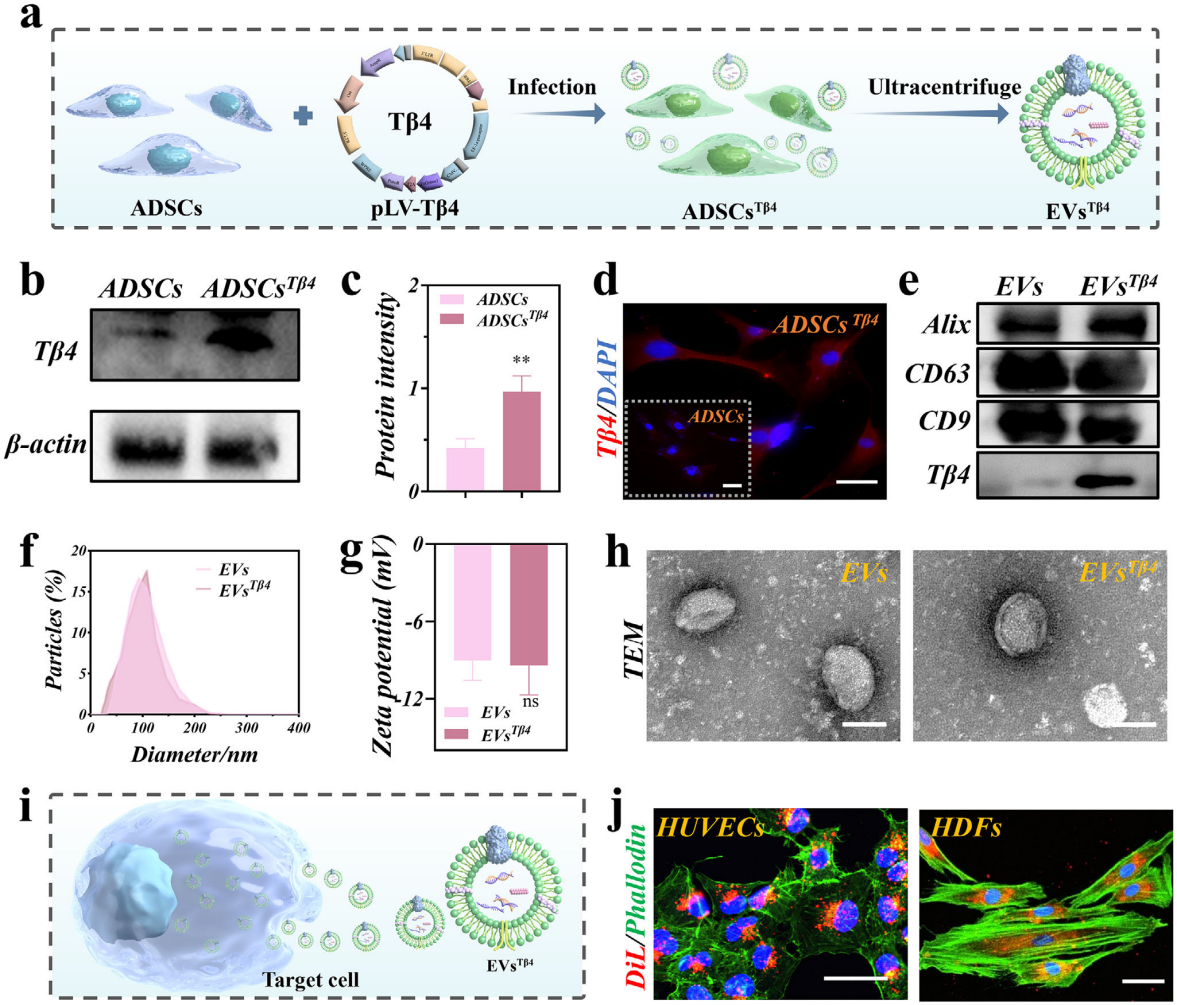
Preparation process and characterization of EVs^Tβ4^. a) Schematic illustration of the preparation of EVs^Tβ4^. (b,c) Tβ4 expression was detected via Western blot b) and quantification analysis c) in ADSCs and ADSCs^Tβ4^. *n* = 3. d) Representative images of Tβ4 were detected via immunofluorescence in ADSCs and ADSCs^Tβ4^. Scale bar = 50 µm. e) EVs markers (Alix, CD63, CD9) and Tβ4 expression were detected via Western blot in EVs and EVs^Tβ4^. f) The average diameter and distribution of EVs and EVs^Tβ4^ was detected via NTA. g) Quantification analysis of zeta potential. *n* = 3. h) TEM images of EVs^Tβ4^ and EVs. Scale bar = 100nm. i) Schematic diagram of EVs^Tβ4^ internalization by target cells. j) Representative images of Dil‐labeled EVs^Tβ4^ (red) were taken up by HUVECs(green) and HDFs (green) in vitro via confocal imaging. Scale bar = 50 µm.

In a typical experiment, lentivirus‐mediated gene transfection and ultracentrifugation were employed to prepare EVs^Tβ4^. Initially, flow cytometry analysis was conducted to characterize ADSCs (Figure S1, Supporting Information). As shown in **Figure** **2**a, Tβ4 was transfected into ADSCs via lentivirus‐mediated techniques and subsequently screened with puromycin to yield ADSCs overexpressing Tβ4 (ADSCs^Tβ4^). To ascertain Tβ4 overexpression in ADSCs^Tβ4^, Western blot assay was conducted. The results indicated a notable increase in Tβ4 expression in ADSCs^Tβ4^ compared to ADSCs (Figure 2b,c). The immunofluorescence findings corroborated the Western blot data (Figure 2d), thereby confirming the successful transfection of Tβ4 into ADSCs. Subsequently, EVs^Tβ4^ isolated via ultracentrifugation demonstrated high enrichment of extracellular vesicles‐specific marker proteins (including CD9, CD63, and Alix) and overexpression of Tβ4 in EVs^Tβ4^ by Western blot analysis (Figure 2e). Furthermore, the particle zeta potential and size of EVs^Tβ4^ were evaluated via nanoparticle tracking analysis (NTA). The diameters of EVs^Tβ4^ were found to range from 50 to 200 nm (Figure 2f), with no notable difference in zeta potential observed between EVs and EVs^Tβ4^ (Figure 2g). Additionally, transmission electron microscopy (TEM) images further confirmed that Tβ4 overexpression did not disrupt the morphology of the EVs (Figure 2h). Collectively, these findings indicate the successful acquisition of Tβ4‐overexpressing ADSC‐EVs.

To demonstrate that EVs^Tβ4^ can infiltrate target cells via direct fusion and endocytosis pathways (Figure 2i). Here, we labeled EVs with cell membrane staining reagent (DiL) dye to confirm cellular uptake of specific EVs. As shown in Figure 2j, DiL‐labeled extracellular vesicles (red) were found within the cytoskeletons of HUVECs (green) and HDFs (green) in confocal microscope images. This observation indicates that EVs^Tβ4^ deliver bioactive substances to HUVECs and HDFs for therapeutic applications. In summary, these results not only demonstrate the successful preparation of the desired EVs^Tβ4^ but also confirmed their cellular uptake, mirroring the physiological uptake of naturally secreted EVs.

**Figure 3:**
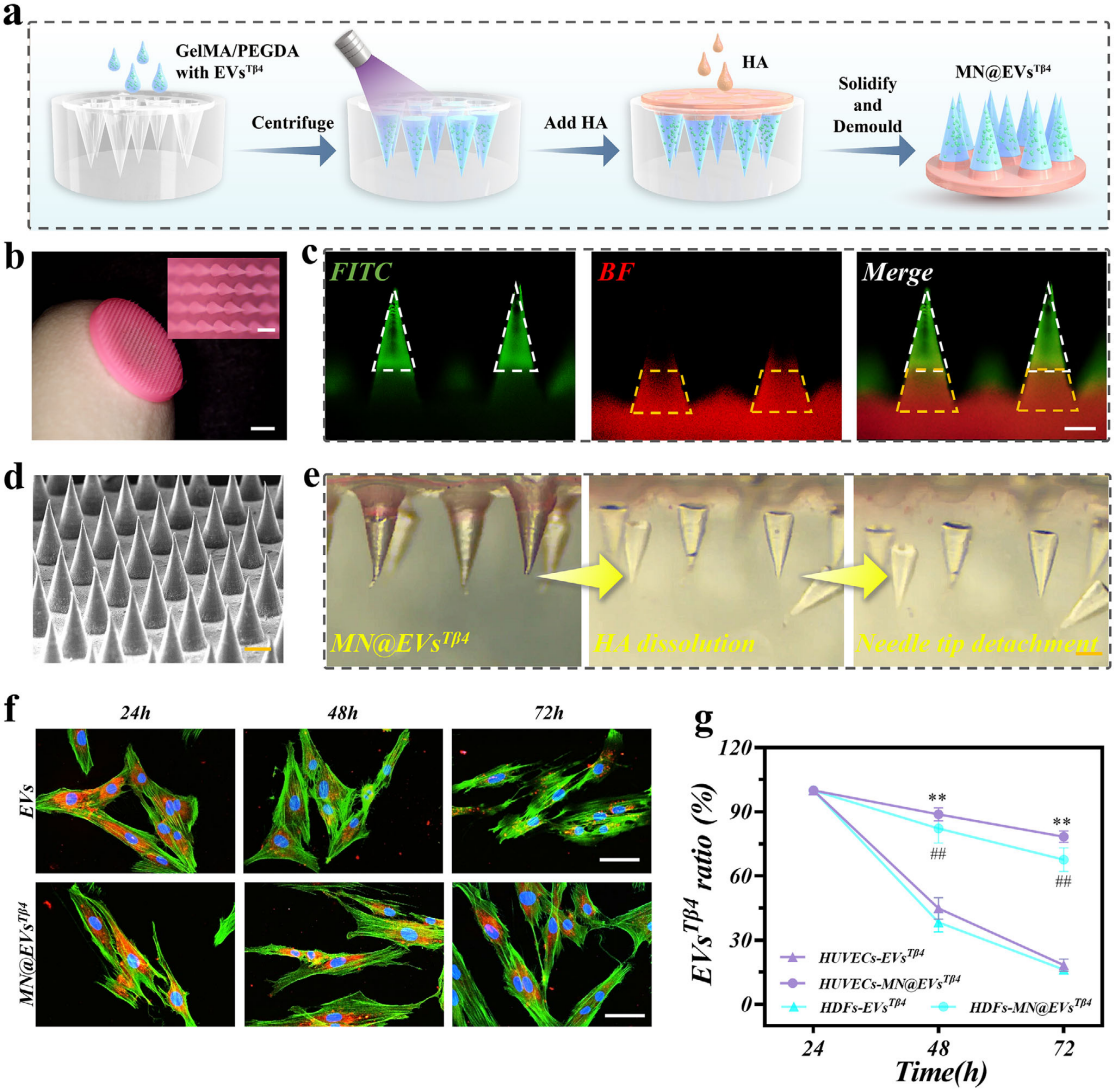
Preparation process and characterization of the MN@EVs^Tβ4^ patch. a) Schematic illustrations of the fabrication of MN@EVs^Tβ4^. b) Micrograph image of MN@EVs^Tβ4^. Scale bar = 3 mm and 200 µm. c) Fluorescence images of MN@EVs^Tβ4^ fabricated by FITC‐labeled GelMA/PEGDA (needle tips) and Rhodamine B‐labeled HA (patch base). Scale bar = 100 µm. d) SEM image of MN@EVs^Tβ4^. Scale bar = 200 µm. e) The images of the complete process before and after the tip detachment in MN@EVs^Tβ4^. Scale bar = 200 µm. f) Typical fluorescence images of HDFs (green) that were co‐incubated with equivalent Dil‐stained EVs^Tβ4^ (red) in free form and MN@EVs^Tβ4^. Scale bar = 50 µm. g) Quantification analysis of Dil‐stained EVs ^Tβ4^ (red) in HUVECs and HDFs at 24, 48, and 72 h. *n* = 3.

For the effective delivery of EVs^Tβ4^ to the wound bed to facilitate tissue repair, a two‐step template replication approach was employed to fabricate the separable microneedle patch (MN@EVs^Tβ4^). As illustrated in **Figure** **3**a, EVs^Tβ4^ were blended into a solution of GelMA and PEGDA, which was then poured into a microneedle template and exposed to UV light to form biodegradable microneedle tips. Subsequently, an HA solution was filled into the base layer and dried. This process resulted in the fabrication of separable microneedle patches with GelMA/PEGDA tips and an HA backing layer. The constituent materials, namely HA, GelMA, and PEGDA, were rigorously analyzed using proton nuclear magnetic resonance (H‐NMR) (Figure S2a–c, Supporting Information) and Fourier transform infrared spectroscopy (FTIR) (Figure S2d–f, Supporting Information), thereby confirming the presence of the intended functional groups and the characteristic absorption peaks of all components. As shown in Figure 3b, MN@EVs^Tβ4^ exhibits a disc shape, measuring 11.0 mm diameter and 2.0 mm height. Scanning electron microscopy (SEM) revealed a conical needle structure with microneedle tips neatly arranged on the HA substrate, each microneedle was featuring 500 µm height and a 300 µm base diameter. (Figure 3d; Figure S3a,b, Supporting Information).

Furthermore, to further investigate the microneedle structure, the needle tips were labeled with Fluorescein 5‐isothiocyanate (FITC), and the base was labeled with Rhodamine B. Fluorescence microscopy corroborated the successful fabrication of the MN@EVs^Tβ4^ patch via the two‐step method (Figure 3c; Figure S3c, Supporting Information). Upon application to the wound area, the HA base layer of the MN@EVs^Tβ4^ patch absorbed wound exudate, dissolving to maintain the wound moisture. In vitro, the successful detachment of the microneedle tips from the base was verified by immersing MN@EVs^Tβ4^ in PBS. The results indicated that the base layer dissolved after absorbing PBS, prompting the microneedle tips to rapidly separate from the patch. Real‐time recordings were made (Video S1, Supporting Information), and photographs were taken before and after detachment (Figure 3e). Moreover, MN@EVs^Tβ4^ demonstrated sufficient mechanical strength, with each microneedle sustaining a radial compression force of ≈0.79 N (Figure S3d, Supporting Information). The radial compression force exceeds the minimum required for successful skin penetration.^[^ ^11^ ^]^ We also investigated the release of EVs^Tβ4^ on microneedles in vitro. The sustained release capacity of MN@EVs^Tβ4^ was demonstrated by the release curve of EVs^Tβ4^ over an 11‐day period in vitro (Figure S3e, Supporting Information).

The penetration capability of microneedles is pivotal for their ability to pierce the skin and effectively deliver therapeutic agents. Consequently, to assess the penetration efficacy of MN@EVs^Tβ4^, the patches were applied to pig skin and agarose gel. As shown in Figure S4a,b (Supporting Information), MN@EVs^Tβ4^ successfully delivered green fluorescent dye to the skin of pig and agarose gel. Hematoxylin and eosin stain (H&E) of pig skin further substantiated that the microneedles successfully penetrated the pig skin without fracturing (Figure S4c, Supporting Information). Moreover, cellular uptake assays confirmed the continuous release of EVs^Tβ4^ from the MN@EVs^Tβ4^. Specifically, free EVs^Tβ4^ were rapidly taken up by target cells and degraded within 72 h, while the release of EVs^Tβ4^ from MN@EVs^Tβ4^ tips remained continuous, with the red fluorescence signal of EVs^Tβ4^ persisting in HUVECs and HDFs even after 72 h (Figure 3f,g; Figure S5, Supporting Information). Collectively, these results demonstrate the successful fabrication of the separable microneedle MN@EVs^Tβ4^ and it sustained continuously release of functional EVs^Tβ4^, thereby achieving the desired therapeutic effect.

**Figure 4:**
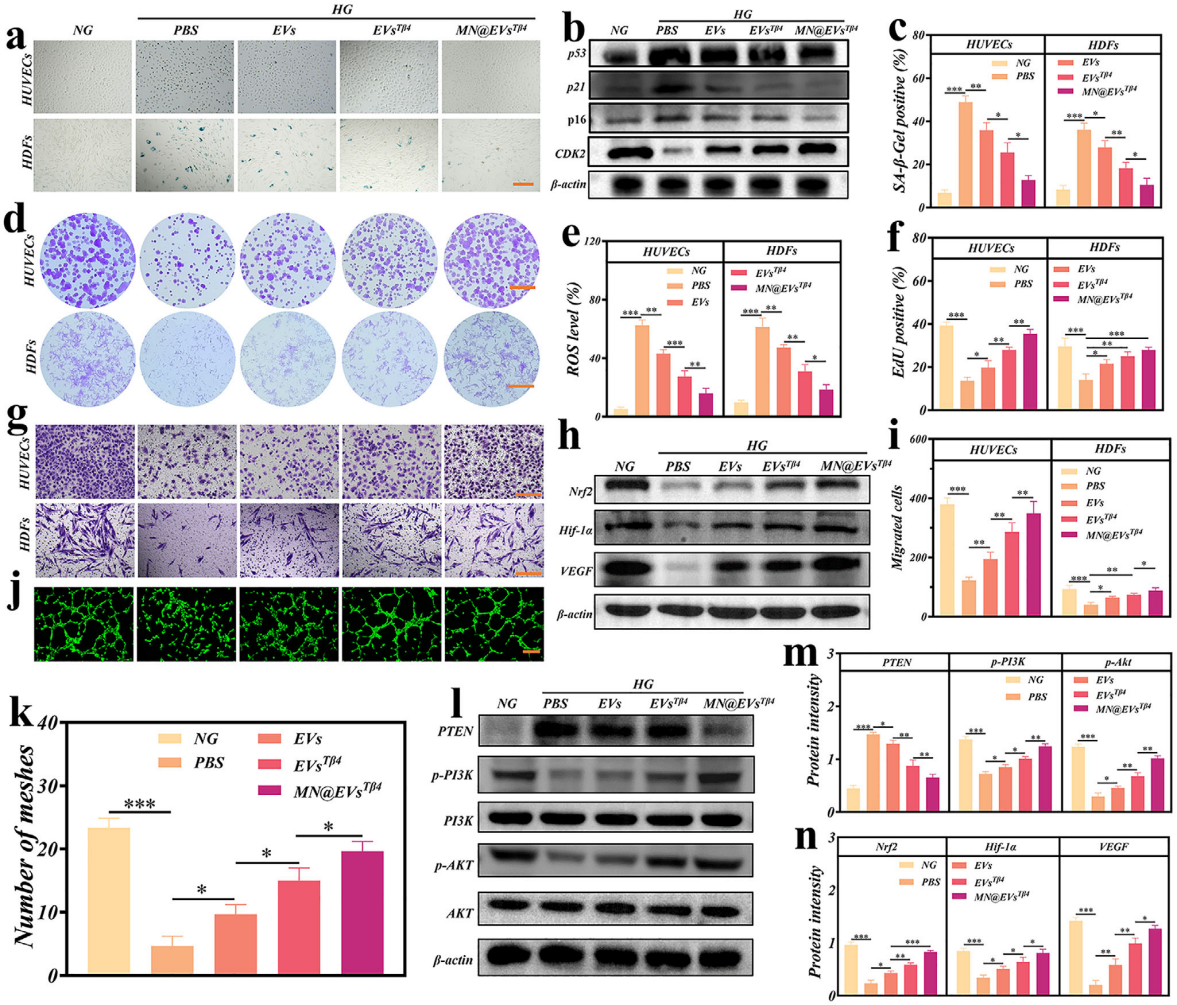
MN@EVs^Tβ4^ rescued cell senescence and optimized cell function in HUVECs and HDFs by PTEN/PI3K/AKT pathway. (a,c) Representative images a) and quantification analysis c) of SA‐β‐gal across different groups. Scale bar = 100 µm, *n* = 3. b) Western blot analysis to assess the levels of p53, p21, p16, and CDK2 in HDFs. d) Representative images of cells colony formation assays. Scale bar = 5 mm. e) ROS levels across different groups were measured by DCFH‐DA. *n* = 3. f) Quantification of EdU staining across different groups. *n* = 3. (g,i) The images g) and quantification analysis i) of transwell assay across different groups. *n* = 3. Scale bar = 100 µm. (h,n) Western blot h) and quantification analysis n) of the protein expression of Nrf2, HIF‐1α, and VEGF across different groups in HUVECs. *n* = 3. (j,k) The images j) and quantification analysis k) of angiogenesis assay across different groups in HUVECs. Scale bar = 100 µm, *n* = 3. (l,m) Western blot l) and quantification analysis m) of PTEN, p‐PI3K and p‐AKT proteins expression in HUVECs across different groups. *n* = 3.

Subsequently, the biocompatibility of MN@EVs^Tβ4^ was assessed by a cell Live/dead staining assay. For this assessment, cells were co‐cultured separately with microneedles (Figure S6, Supporting Information). The results indicated that MN@EVs^Tβ4^ exhibited negligible cytotoxicity toward both HUVECs and HDFs (Figure S7, Supporting Information). To investigate the potential of MN@EVs^Tβ4^ in alleviating high glucose (HG)‐induced cellular senescence and enhancing cell function in HUVECs and HDFs, we observed that following HG (33mm) stimulation, the expression of β‐galactosidase (SA‐β‐gal) was notably higher in HG group compared to the normal glucose group (NG), in accordance with prior studies.^[^ ^12^ ^]^ Following treatment with EVs, EVs^Tβ4^, and MN@EVs^Tβ4^, a marked reduction in the number of SA‐β‐gal positive cells was observed, with the greatest decrease seen in the MN@EVs^Tβ4^ group (**Figure** **4**a,c).

Furthermore, the anti‐senescence effects of MN@EVs^Tβ4^ were investigated by evaluating the levels of senescence‐related p16, p53, and p21 proteins in HG‐stimulated HUVECs and HDFs. p21, in conjunction with the cyclin‐dependent kinase (CDK2), regulates the cell cycle, with their expression being inversely correlated. Consequently, CDK2 expression was also evaluated. The results revealed that treated with EVs or EVs^Tβ4^ led to a moderate downregulation of p53, p21, and p16, while treatment with MN@EVs^Tβ4^ resulted in a more pronounced reduction (Figure 4b; Figure S8a‐i, Supporting Information). In contrast, CDK2 expression exhibited an opposite trend to that of p21. In summary, MN@EVs^Tβ4^ effectively alleviates HG‐induced cellular senescence in HUVECs and HDFs.

Previous research has established that excessive intracellular reactive oxygen species (ROS) production speeds up cellular senescence in the diabetic microenvironment.^[^ ^13^ ^]^ In this study, we explored whether MN@EVs^Tβ4^ could effectively neutralize intercellular ROS. Treatment with MN@EVs^Tβ4^ markedly diminished ROS production, while administration of EVs or EVs^Tβ4^ only moderately alleviated ROS levels, as indicated by the green fluorescence in Figure S9a (Supporting Information), Figure 4e. Flow cytometry analysis revealed that HUVECs and HDFs co‐cultured with MN@EVs^Tβ4^ were protected from ROS‐induced damage, consistent with the results of ROS staining (Figure S9c,d, Supporting Information).

The proliferative capacity under various treatments was further assessed using EdU staining. A greater proportion of EdU‐positive cells, marked by red fluorescence, was observed in EVs, EVs^Tβ4^, and MN@EVs^Tβ4^ groups, with the highest proportion found in MN@EVs^Tβ4^ group (Figure 4f; Figure S9b, Supporting Information). Additionally, a colony formation assay confirmed that MN@EVs^Tβ4^ had the most pronounced effect on the proliferation of HUVECs and HDFs (Figure 4d; Figure S9e, Supporting Information). The effects of MN@EVs^Tβ4^ on cell migration were evaluated using transwell and scratch assays. As shown in Figure 4g,i, all treatment groups exhibited a notable increase in migrating cells compared to PBS group, with the MN@EVs^Tβ4^ group exhibiting the highest migration. Similarly, in the scratch assay, EVs, EVs^Tβ4^, and MN@EVs^Tβ4^ significantly enhanced gap closure compared to PBS group. Moreover, MN@EVs^Tβ4^ group displayed a larger migration area than the EVs or EVs^Tβ4^ groups (Figure S10a,b, Supporting Information). In conclusion, MN@EVs^Tβ4^ significantly enhanced the migration capacity of HG‐induced HUVECs and HDFs.

Next, the effect of MN@EVs^Tβ4^ on the angiogenic properties of HUVECs was investigated. As shown in Figure 4j,k, MN@EVs^Tβ4^‐treated HUVECs exhibited the most robust tube formation, displaying the highest number of capillary branches and well‐formed tubular structures. Thus, MN@EVs^Tβ4^ demonstrated the strongest angiogenic potential in HG‐induced HUVECs. Previous research has indicated that Tβ4 plays an important role in regulating PTEN/PI3K/AKT pathway, a pivotal pathway in the modulation of cellular senescence.^[^ ^14^ ^]^ Based on this, we hypothesized that MN@EVs^Tβ4^ alleviates cell senescence by modulating PTEN/PI3K/AKT. Indeed, Western blot analysis revealed that the cells treated with MN@EVs^Tβ4^ exhibited lower PTEN levels compared to those treated with EVs or EVs^Tβ4^. Following the inhibition of PTEN expression, the p‐PI3K and p‐AKT were subsequently activated (Figure 4l,m; Figure S11a,b, Supporting Information). According to the literature, the inhibition of PTEN not only promotes cell growth and migration but also increases the expression of Nrf2, which mitigates excess ROS and rescues ROS‐induced senescence in certain pathological conditions.^[^ ^15^ ^]^ Consequently, we assessed the protein levels of Nrf2 and functional proteins (HIF‐1α, VEGF) in HUVECs. As shown in Figure 4h,n, treatment with EVs or EVs^Tβ4^ moderately restored the expression of Nrf2, while treatment with MN@EVs^Tβ4^ significantly restored Nrf2 expression. Similar results were observed for HIF‐1α and VEGF expression.

Moreover, the levels of Nrf2 and functional proteins (COL1A1 and α‐SMA) in HDFs mirrored this trend, signifying enhanced collagen secretion and contribution to wound healing (Figure S11c,d, Supporting Information). Thus, activation of the PI3K/AKT pathway upregulates Nrf2 expression to counteract excessive ROS and increases the expression of HIF‐1α and VEGF to foster angiogenesis. Concurrently, the upregulation of COL1A1 and α‐SMA expression promoted collagen deposition. In summary, MN@EVs^Tβ4^ may rescue cell senescence and improve cell function by targeting PTEN/PI3K/AKT pathway, thereby providing an effective therapeutic strategy for diabetic chronic wounds.

**Figure 5:**
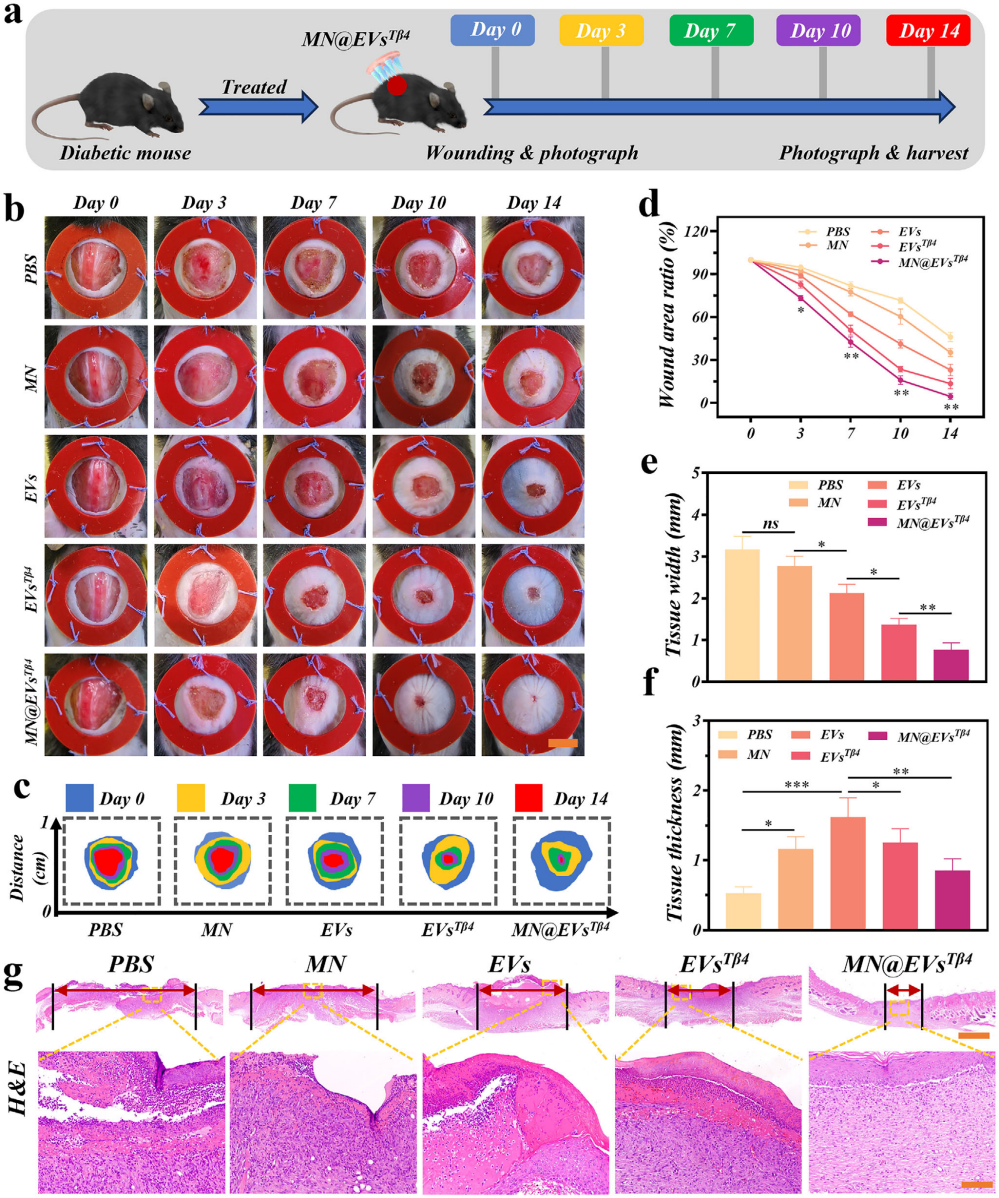
MN@EVs^Tβ4^ enhanced wound healing in diabetic mice in vivo. a) Schematic diagram illustrating the experimental procedure for wound healing studies in diabetic mice. (b,c) The photographs b) and optical images c) showing wound contraction on days 0, 3, 7, 10, and 14. Scale bar = 5 mm. d) The residual wound area at different time points across the groups. *n* = 5. (e,f) The analysis of tissue thickness and tissue width across different groups. *n* = 5. g) H&E staining across different groups of the diabetic wound tissue. Scale bar = 1 mm and 100 µm.

Based on the biocompatibility of MN@EVs^Tβ4^ patches and the effects on the functional cells in vitro, the potential of MN@EVs^Tβ4^ to enhance diabetic wound healing was further evaluated in vivo. Initially, a circular wound with a 1 cm diameter was created on the dorsal region of STZ‐induced diabetic mice. Throughout the process, wounds were observed and photographed at 0, 3, 7, 10, and 14 days (**Figure** **5**a). Macroscopic wound analysis revealed that MN@EVs^Tβ4^ group exhibited the smallest wound area throughout the period (Figure 5b,c). In quantitative analysis, the MN@EVs^Tβ4^ group exhibited the highest wound closure rate, followed by EVs^Tβ4^, EVs, blank microneedles(MN), and PBS group (Figure 5d).

These findings were further confirmed by H&E staining of tissue sections from each group. In MN@EVs^Tβ4^ treated group, a newly formed, well‐organized epidermis was observed, characterized by regenerated skin appendages and complete epithelial reformation. The subsequent groups, in order of completeness, were EVs^Tβ4^, EVs, and MN, with delayed epithelial reformation observed in the PBS group (Figure 5g). Upon comparison of wound tissue thickness and width through H&E staining, it was found that MN@EVs^Tβ4^ group had an epidermal thickness closest to normal skin, with relatively intact skin layers (Figure 5e,f). Additionally, the MN@EVs^Tβ4^ group exhibited the smallest wound width, followed by EVs^Tβ4^, EVs, MN, and the poorest healing quality in PBS group. These results demonstrate that MN@EVs^Tβ4^, EVs^Tβ4^, EVs, and MN groups all effectively promoted epithelial reformation, with the most pronounced effect observed in MN@EVs^Tβ4^. Overall, these results indicated that MN@EVs^Tβ4^ exhibited the most significant effect in promoting epithelial transformation in diabetic wounds.

**Figure 6:**
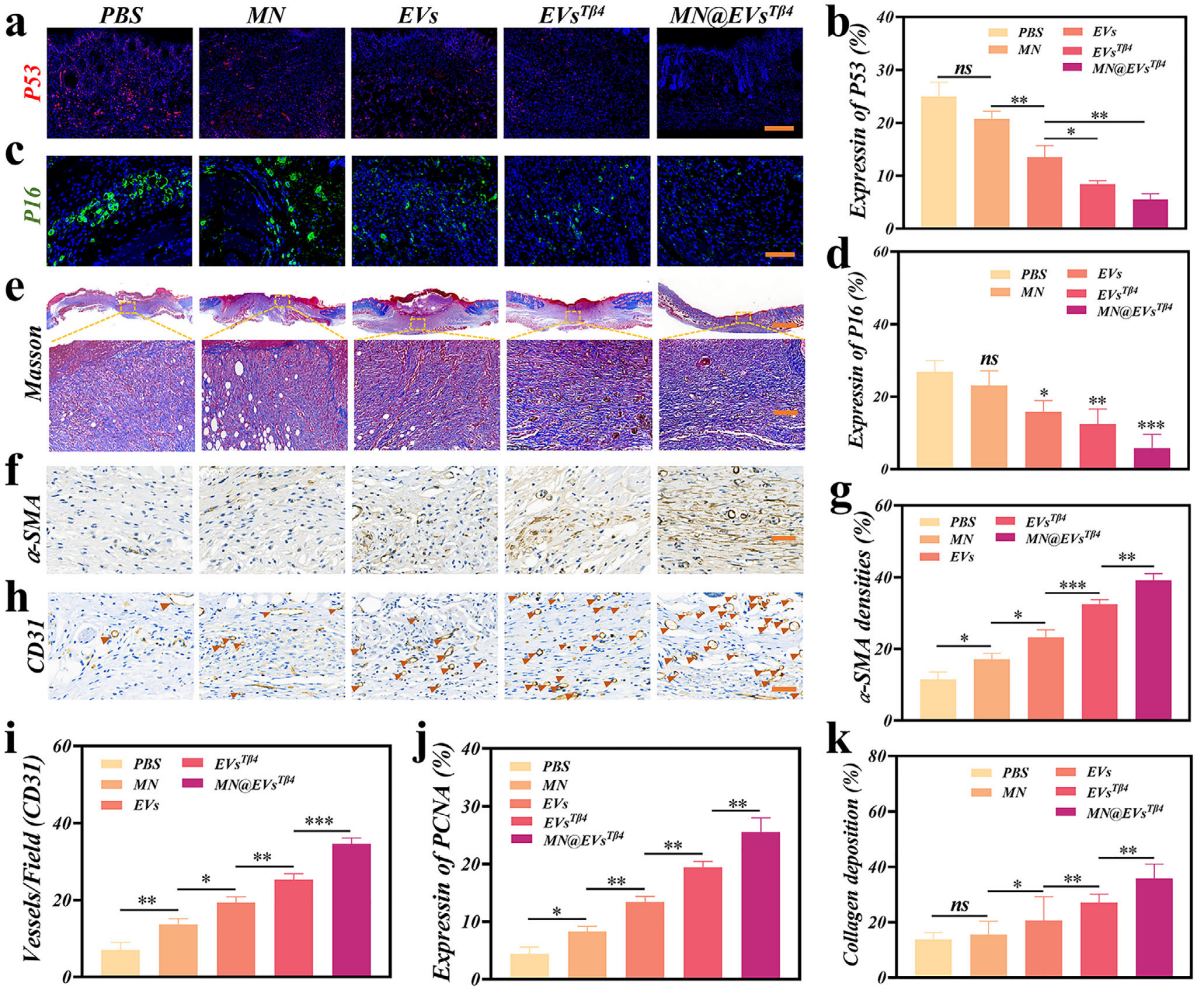
MN@EVs^Tβ4^ promotes collagen deposition and angiogenesis via reducing senescence and enhancing cell proliferation in vivo. (a,b) Immunofluorescence staining a) and quantification analysis b) of p53 in tissues in different treatments. Scale bars = 100 µm, *n* = 3. (c,d) Immunofluorescence staining c) and quantification analysis d) of p16 in tissues in different treatments. Scale bars = 100 µm, *n* = 3. e) Masson's trichrome staining of tissues across different groups. Scale bars = 1 mm and 100 µm. (f,g) Immunohistochemical staining f) and quantification analysis g) of α‐SMA in tissues across different groups. Scale bars = 100 µm, *n* = 3. (h,i) Immunohistochemical staining h) and quantification analysis i) of CD31 in tissues across different groups. Scale bars = 100 µm, *n* = 3. j) Quantification analysis of PCNA expression in tissues across different treatments. *n* = 3. k) Quantification analysis of collagen deposition. *n* = 3.

Additionally, to further investigate the effects of MN@EVs^Tβ4^ on senescence and proliferation in diabetic wound skin repair cells, the levels of p53, p16, p21, and PCNA were assessed. Immunofluorescence and immunohistochemical staining revealed elevated expression of p53, p16, and p21 in PBS group, indicating that the skin cells at the diabetic wound site were in a state of senescence. In contrast, the levels of p53, p16, and p21 were reduced in MN@EVs^Tβ4^, EVs^Tβ4^, and EVs groups, suggesting an improvement in the cellular senescence state. Notably, compared to the free EVs^Tβ4^, MN@EVs^Tβ4^ exhibited lower expression levels of p53, p16, and p21 (**Figure** **6**a–d; Figure S12a,b, Supporting Information). Conversely, immunofluorescence analysis revealed the highest PCNA expression in MN@EVs^Tβ4^ group, indicating active proliferation of skin repair cells (Figure S13, Supporting Information; Figure 6j).

To further investigate the role of HDFs in wound repair, we evaluated collagen deposition in wound tissues from each group by Masson's staining. The result shown that collagen deposition was significantly higher in the MN@EVs^Tβ4^, EVs^Tβ4^, and EVs groups compared to the PBS and MN groups (Figure 6e,k). In the MN@EVs^Tβ4^ group, collagen exhibited a more organized and mature distribution. Similarity, immunohistochemical staining was performed to assess α‐SMA expression in wound tissues. As shown in Figure 6f,g, the MN@EVs^Tβ4^ group exhibited the highest α‐SMA expression, followed by EVs^Tβ4^, EVs, and MN groups. This indicates that a substantial number of HDFs were transformed into myofibroblasts in wound tissues, significantly accelerating the process of the diabetic wound healing. Consistent with the immunohistochemical and Masson's staining results, Western blot analysis revealed that the MN@EVs^Tβ4^ group exhibited the highest levels of COL1A1 and α‐SMA expression (Figure S15a–c, Supporting Information).

Immunohistochemical staining was also conducted to evaluate vascular formation at the wounds site using the endothelial cell marker CD31. Interestingly, wounds treated with MN@EVs^Tβ4^ exhibited the highest CD31 expression, followed by EVs^Tβ4^, EVs, MN, and PBS groups (Figure 6h,i). Furthermore, we conducted immunofluorescence staining for α‐SMA and CD31 in wound tissues, and the findings were consistent with those from immunohistochemical analysis (Figure S14, Supporting Information). Similarly, Western blot analysis was performed to assess VEGF, another endothelial cell marker, and the results were consistent with the immunohistochemical findings, with MN@EVs^Tβ4^ showing the highest VEGF expression (Figure S15a,d, Supporting Information). H&E staining of the organs from each group of mice showed that the MN was safe for mice (Figure S16, Supporting Information). Collectively, MN@EVs^Tβ4^ accelerates collagen deposition and angiogenesis in diabetic wounds via reducing cellular senescence and boosting proliferation.

## 3 Conclusion

In conclusion, we reported a novel microneedle patch MN@EVs^Tβ4^ loaded with Tβ4‐engineered ADSC‐derived EVs. This innovative patch features GelMA/PEGDA as the microneedle tips and HA as the microneedle base. The base absorbed exudate, dissolved to protect the wound, and facilitated the detachment of microneedle tips to deliver encapsulated EVs^Tβ4^ to the wound site for the treatment of diabetic chronic wounds. In vitro, MN@EVs^Tβ4^ continuously releases EVs^Tβ4^, which were subsequently internalized by target cells. MN@EVs^Tβ4^ rescued HUVECs and HDFs cellular senescence and enhanced cellular function via targeting PTEN/PI3K/AKT pathway. In vivo, the sustained release of EVs^Tβ4^ from MN@EVs^Tβ4^ at the wound site enhanced its therapeutic efficacy. It suppressed the expression of p16, p21, and p53 in wound tissues, mitigating cellular senescence and promoting cellular proliferation to enhance angiogenesis and collagen deposition, thereby accelerating the healing of chronic wounds. This novel engineered extracellular vesicle‐based MN patch holds promise for practical applications in treating diabetic wounds and other chronic wounds.

## 4 Experimental Section

### Materials

The 2‐hydroxy‐2‐methylpropiophenone (HMPP) was obtained from Sigma–Aldrich. The Gelatin methacryloyl (GelMA) was self‐prepared. Hyaluronic acid (HA) and polyethylene glycol diacrylate (PEGDA) was purchased from Aladdin, Shanghai, China. The GFP‐Tβ4 lentivirus was manufactured by Yanming Biotechnology, Shenzhen, China. The Antibodies of Tβ4, ALix, CD63, CD9, CD31, p21, p16, HIF‐1α, COL1A1, and α‐SMA were from Proteintech, Wuhan, China. The Antibodies of VEGF, p53, CDK2, PTEN, Pi3k, p‐Akt, p‐Pi3k, and Akt was from HUABIO, Hangzhou, China. The Live/Dead Cell Stain Kit was sourced from Beyotime Biotechnology, China. Fetal bovine serum (FBS) was brought from Gibco. CD73, CD105, CD90, CD44, CD34 and CD45 were purchased from BD Pharmingen. The ADSCs were from ScienCell (Catalog #7510) were utilized. The HDFs and HUVECs were obtained from the Bank of cell (Shanghai, China). C57/B6 mice were from Sellingmice (Nanjing, China) Biotechnology Co., Ltd.

### Cell Culture and Cellular Uptake

ADSCs were transfected with lentivirus to overexpress Tβ4. The lentivirus infection was assisted by Polybrene, and puromycin was added for screening. ADSCs were cultured in DMEM/F‐12 complete culture medium. HUVECs and HDFs were cultured in 5.5 mm D‐glucose (NG) or 33 mm D‐glucose (HG) DMEM complete culture medium. For cell uptake assay, EVs^Tβ4^ were stained with Dil (red, Yeasen Biotechnology, Shanghai). The HUVECs and HDFs were inoculated in 24‐well plates and then incubated with EVs^Tβ4^ labeled Dil (10 µg mL^−1^) for 6 h. After rinsing, cells were cultured with paraformaldehyde at 25 °C for 20 min. Then, cells were stained with 4 µg mL^−1^ SF488 Phalloidin (Green) (Solarbio, China, CA1640) for 20 min and finally stained with DAPI for 10 min. Cellular uptake of EVs^Tβ4^ was obtained using confocal microscopy (Nikon, FV 3000). For β‐galactosidase assay, the expression of SA‐β‐Gal in HUVECs and HDFs was observed in different groups using SA‐β‐Gal (C0602, Beyotime, China,) kit, following the instructions.

### Extraction, Purification, and Identification of ADSC Derived EVs with Tβ4 Overexpression

To extract EVs^Tβ4^, ADSC^Tβ4^ was cultured in a culture medium free of exosomes. The medium was centrifuged at 300×g and 2000×g for 10 min, followed by ultracentrifugation at 10 000×g for 30 min. The cells, membranes, and debris were discarded. The supernatant was then filtered through a 0.22 µm filter. Finally, the supernatant was then centrifuged at 120000×g at 4 °C for 1.5 h. The pellet was resuspended with PBS, and then subjected to another round of centrifugation at 120000×g for 1.5 h. EVs^Tβ4^ were obtained. The morphology and diameter of EVs^Tβ4^ were observed by TEM.

### Fabrication and Characterization of MN@EVs^Tβ4^ Patch

The fabrication of the MN@EVs^Tβ4^ was carried out using polydimethylsiloxane (PDMS) microneedle molds. Specifically, 60 µL of a solution containing 1 wt.% HMPP, 15 wt.% GelMA, 5% PEGDA, and 200 µg of EVs^Tβ4^ was added to the PDMS needle cavities. The PDMS with mixture was centrifuged at 1000×g for 6 min. The excess solution was then removed via a micropipette tip. Subsequently, the mixture was exposed to 365 nm UV irradiation for 60 s to form the microneedle tips. Then, 200 µL of a solution containing 20 wt.% HA was added to the PDMS. This solution was subsequently deposited to fill the substrate. To observe the microneedles, the tips were labeled with FITC, and HA was fluorescently labeled with Rhodamine B. Fluorescent MN@EVs^Tβ4^ was then prepared as described above. The samples were observed using SEM. Images of MN@EVs^Tβ4^ were captured by fluorescence microscope.

### The Release Test of EVs^Tβ4^

To determine the release kinetics of Dil‐labeled extracellular vesicles from MN@EVs^Tβ4^, Dil‐labeled MN@EVs^Tβ4^ were immersed in a 24‐well plate with PBS, and the supernatant was collected daily for 11 days. The fluorescence intensity of released EVs^Tβ4^ was evaluated at 570 nm via microplate reader. In vitro, Dil‐labeled EVs^Tβ4^ released from MN@EVs^Tβ4^ patches were internalized by target cells and visualized using a confocal microscope.

### Cell Migration Experiment

For cell scratch assay, HG induced HUVECs and HDFs were cultured in 6‐well plates and cultured until they nearly confluent. After an 8‐hour starvation period, cells were scraped by a 200 µL pipette tip. Fresh culture medium with 1% FBS was added to the wells, and the cells were coculture with PBS, EVs, EVs^Tβ4^, or MN@EVs^Tβ4^. The control group consisted of HUVECs and HDFs cells culture with normal glucose medium. At 0 and 24 h post‐scratching, the cells were cultured with calcein‐AM for 15 min and observed via a fluorescence microscope.

The migration ability of HG‐induced HUVECs and HDFs was assessed by Transwell chambers (Corning, USA). A specified number of HUVECs and HDFs were cultured in upper chamber of Transwell plates, while the lower chamber with PBS, EVs, EVs^Tβ4^, or MN@EVs^Tβ4^. The cells were cultured for 24 h, then the cells were cultured with paraformaldehyde for 15 min, cultured with crystal violet for 20 min, and observed by microscope.

### Proliferation Assay

EdU staining was performed by the EdU kit (Beyotime, China). HG‐induced HUVECs and HDFs were plated in 24‐well plates and separately cocultured with PBS, EVs, EVs^Tβ4^, or MN@EVs^Tβ4^ for 24 h. The control group consisted of HUVECs and HDFs cells culture with normal glucose medium. The cells were cocultured with EdU for 2 h. After fixation with paraformaldehyde for 20 min and permeabilization with 0.4% Triton solution for 10 min, the cells were then cultured for 30 min with the Click reaction solution, followed by DAPI staining for 10 min.

For the plate cloning experiment, logarithmic‐phase HG‐induced HUVECs and HDFs were plated at a density of 300 cells in 6‐well plates per well and treated with PBS, EVs, EVs^Tβ4^, or MN@EVs^Tβ4^, respectively. The control group consisted of cells culture with normal glucose medium. The DMEM medium was replaced every 3 days. The cells were continuously cultured for 9–14 days. The culture was terminated when clones appear in a Petri dish. The cells were cultured for 15 min with paraformaldehyde, then washed with PBS. The crystal violet solution was cultured to the wells for 20–30 min, followed by PBS washing and air‐drying at room temperature. Images were taken, and then counted the number of cell clusters.

### Tube Formation Test

An endothelial tube formation test was conducted to assess angiogenesis in each treatment group. HG‐induced HUVECs were seeded in Matrigel‐coated chambers and cultured until the cells adhered to the surface. The cells were then incubated with PBS, EVs, EVs^Tβ4^, or MN@EVs^Tβ4^. The control group consisted of cells culture with normal glucose medium. After 8 h, HUVECs were labeled with calcein‐AM. The treated cells were captured via inverted fluorescence microscope.

### Antioxidant Efficiency

HG‐induced HUVECs and HDFs were plated in 24‐well plates and exposed to high glucose to induce ROS damage. The cells were then treated with PBS, EVs, EVs^Tβ4^, or MN@EVs^Tβ4^ for 24 h. The control group consisted of cells culture with normal glucose medium. HUVECs and HDFs were stained with DCFH‐DA, and fluorescence images were captured with the microscope. Flow cytometry was performed to analyze the number of positive cells using the same method.

### Western Blot Analysis

HG‐induced HUVECs and HDFs were cultured cultured until they reached 75% confluence. The cells were then incubated with PBS, EVs, EVs^Tβ4^, or MN@EVs^Tβ4^ for 24 h. The control group was treated with normal glucose medium. The protein was extracted by the Protein Extraction Kit (Solarbio), and Western blot analysis was conducted as previously described.^[^ ^16^ ^]^

### In Vivo Wound Healing

To induce diabetic mice wound model, the procedure was performed as described in our previous work.^[^ ^17^ ^]^ Briefly, the mice were fasted for 12 h and then injected for 5 consecutive days with STZ (60 mg kg^−1^). When the blood glucose of the mice was kept above 16.7 mmol L^−1^, the wound was created on dorsal surface of the mice, which were then divided into 5 groups: PBS, MN, EVs, EVs^Tβ4^, and MN@EVs^Tβ4^ groups. Each group received the corresponding treatment, and photographs were taken on days 0, 3, 7, 10, and 14. Residual diabetic wound tissues from each group were collected for H&E, Masson, immunohistochemistry (p21, CD31, α‐SMA), and immunofluorescence staining (p53, PCNA, p16, CD31, α‐SMA) to monitor the wound healing process. These included measurements of regenerated granulation tissue formation, skin thickness, collagen deposition, wound width, new blood vessel formation, and cellular senescence in the wound tissues. The animals were performed in accordance with the guidelines of the Nanjing First Hospital Animal Ethics Committee (DWSY‐24165623).

### Statistical Analysis

Statistical analysis was assessed using Student's t‐test and two‐way ANOVA in GraphPad Prism 8 software. ^*^ *p* indicated significance < 0.05, ^**^ *p*, ^##^p indicated significance < 0.01, and ^***^ *p* indicated significance < 0.001, while *p* > 0.05 was deemed non‐significant (ns).

## Conflict of Interest

The authors declare no conflict of interest.

## Author Contributions

Y.D. performed investigation, conceptualization, methodology, validation, wrote—original draft, wrote—reviewed & edited, and funding acquisition. J.W. performed methodology, wrote—reviewed & edited. J.L. performed methodology, wrote—reviewed & edited. Y.C. performed methodology, wrote—reviewed & edited. S.Z. performed methodology, and validation. Y.Z. performed supervision, wrote—reviewed & edited. M.Z. performed conceptualization, supervision, wrote—reviewed & edited, and funding acquisition. Y.Z. Performed conceptualization, project administration, and supervision.

## Supporting information



Supporting Information

Supplemental Video 1

## Data Availability

Research data are not shared.
