# Supplementary material for: Tβ4‐Engineered ADSC Extracellular Vesicles Rescue Cell Senescence Through Separable Microneedle Patches for Diabetic Wound Healing
Source: Adv Sci (Weinh). 2025 Apr 25;12(26):2505009. doi: 10.1002/advs.202505009 (PMC12245110; doi:10.1002/advs.202505009)
Supplement: Supplementary file 1 — Supporting Information [file ADVS-12-2505009-s002.docx]

**Supplementary Information**

**Tβ4-engineered ADSC extracellular vesicles rescue cell senescence through separable microneedle patches for diabetic wound healing**

Youjun Ding^1,3^, Jinglin Wang^1^, Jiaye Li, Yi Cheng^1^, Shuyin Zhou^1^, Yepeng Zhang^1,^*, Yuanjin Zhao^1,2^* and Min Zhou^1,^*

^1^ Department of Vascular Surgery, Cardiovascular medical center, Nanjing Drum Tower Hospital, Clinical College, Jiangsu University, Nanjing, 210002, China

^2^ State Key Laboratory of Bioelectronics, School of Biological Science and Medical Engineering, Southeast University, Nanjing, 210096, China

^3^ Department of Emergency Surgery, The Fourth Affiliated Hospital of Jiangsu University (Zhenjiang Fourth People’s Hospital), Zhenjiang, 212002, China.

*Corresponding authors:

Email: Yepeng Zhang (220153906@seu.edu.cn); Yuanjin Zhao ([yjzhao@seu.edu.cn](mailto:yjzhao@seu.edu.cn)); Min Zhou (zhouminnju@nju.edu.cn)


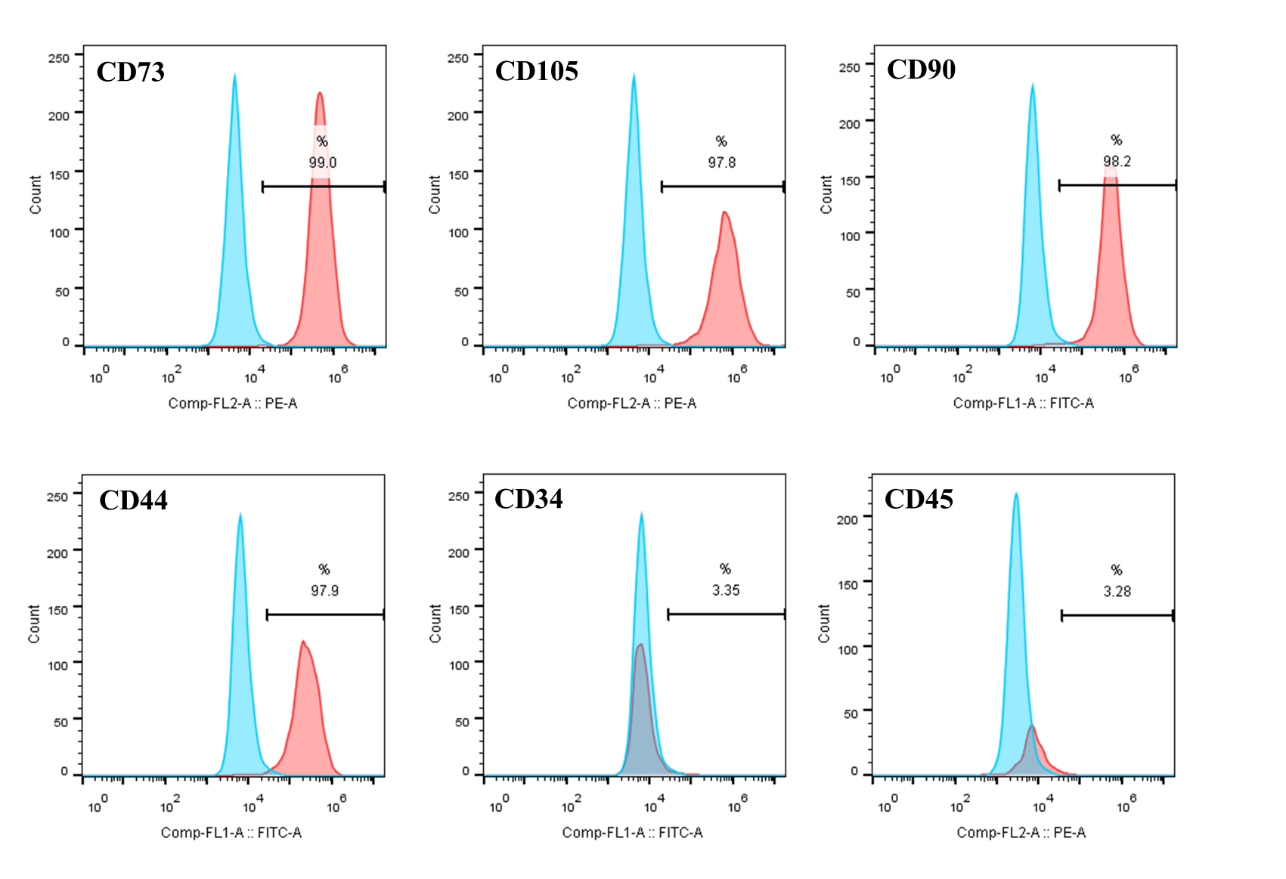


**Figure S1.** Flowcytometric analysis of cell surface markers of the ADSCs.


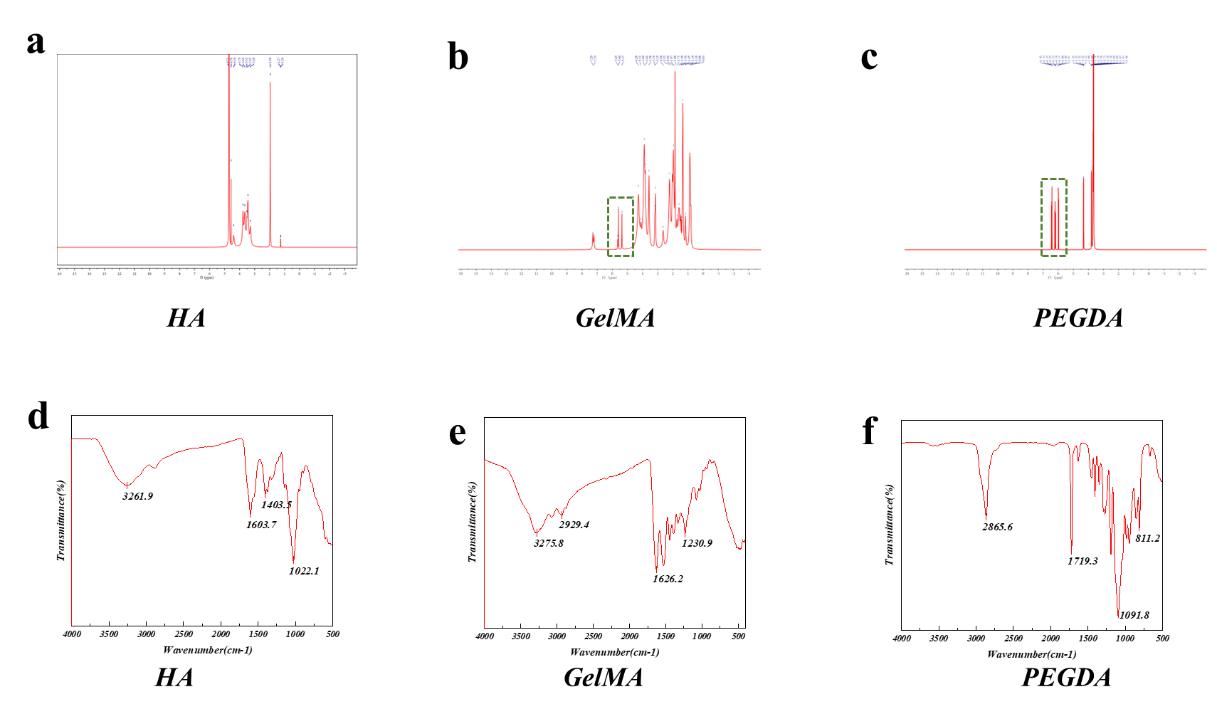


**Figure S2.** (a-c) ^1^H NMR spectrum of HA (a), GelMA (b), PEGDA (c). (d-f) FTIR spectra of HA(d), GelMA (e), PEGDA (f).


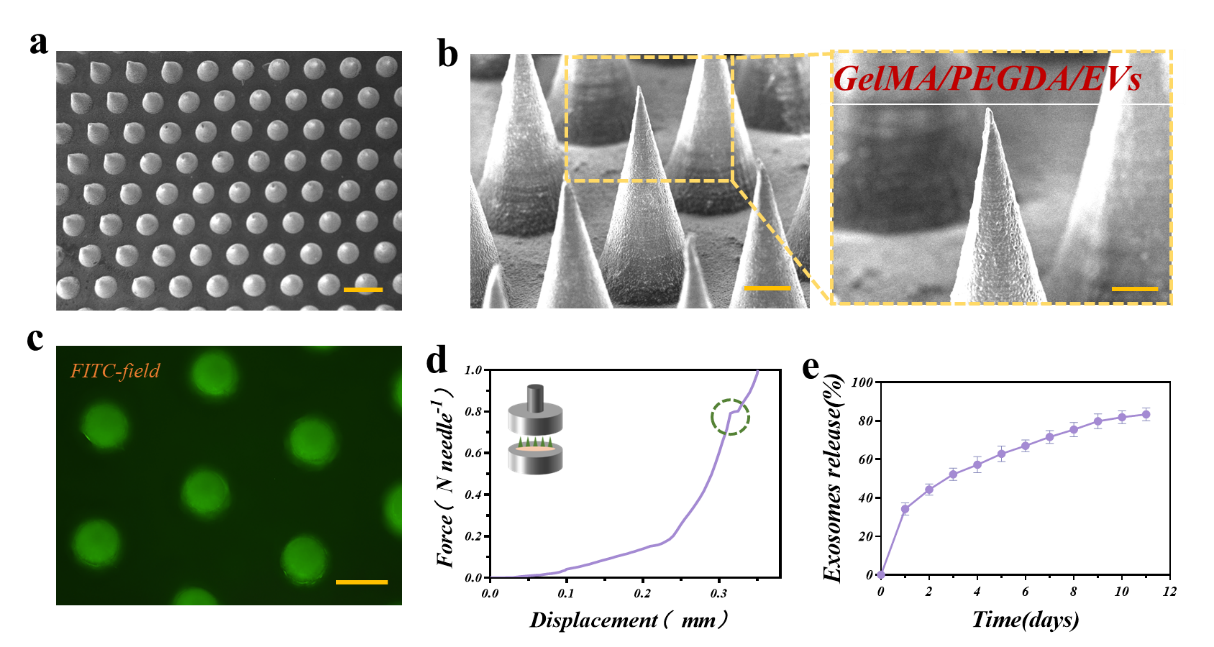


**Figure S3.** (a) SEM images of MN@EVs^Tβ4^. Scale bar = 500μm. (b) SEM magnified images of MN@EVs^Tβ4^. Scale bar = 100μm and 50μm. (c) Representative images of the front view of the MN@EVs^Tβ4^ patch were captured using fluorescence microscope. Scale bar = 100μm. (d) The force−displacement curves of MN@EVs^Tβ4^. (e) Corresponding release kinetics of EVs of MN@EVs^Tβ4^ in PBS. n = 3.


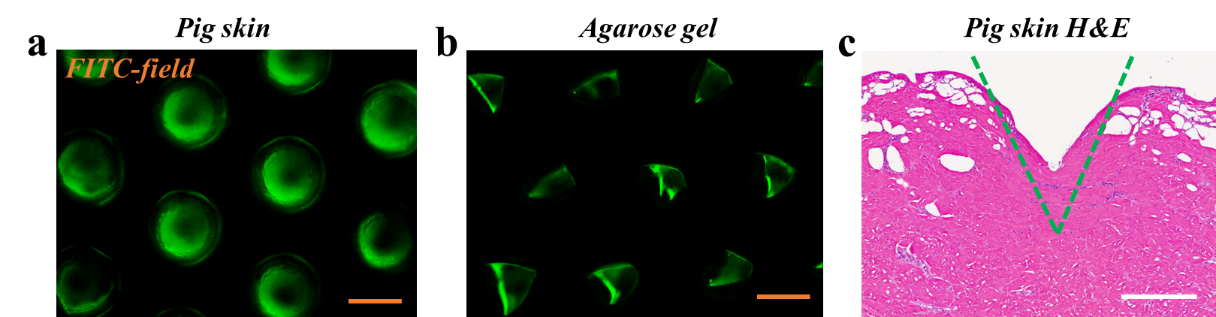


**Figure S4.** (a, b) Representative image of fluorescence-field pig skin (a) and agarose gel (b) after being punctured by MN@EVs^Tβ4^. Scale bar = 200μm. (c) H&E staining of porcine skin after pierced by MN@EVs^Tβ4^. Scale bar = 200μm.

**
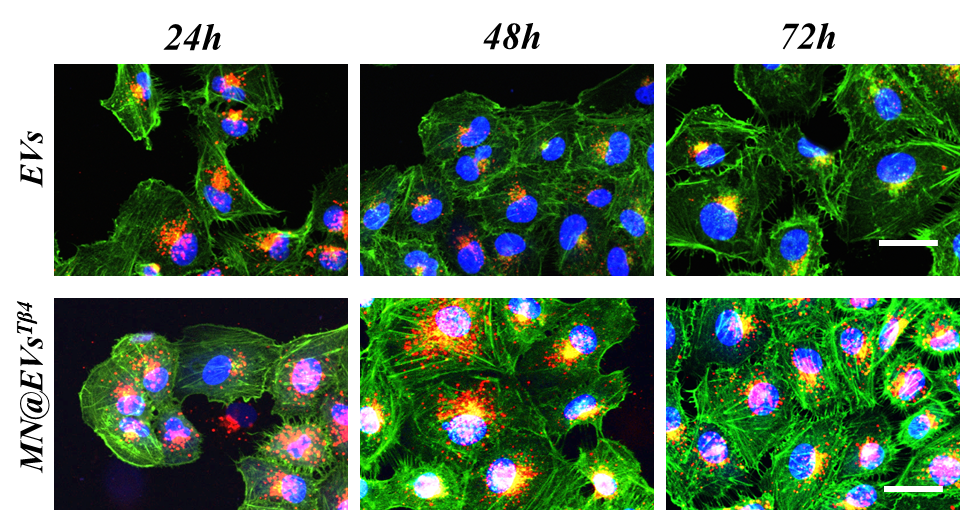
**

**Figure S5.** Typical fluorescent images of HUVECs that were co-incubated with equivalent Dil-stained EVs^Tβ4^ (red) in free form and MN@EVs^Tβ4^. Scale bar = 50μm.


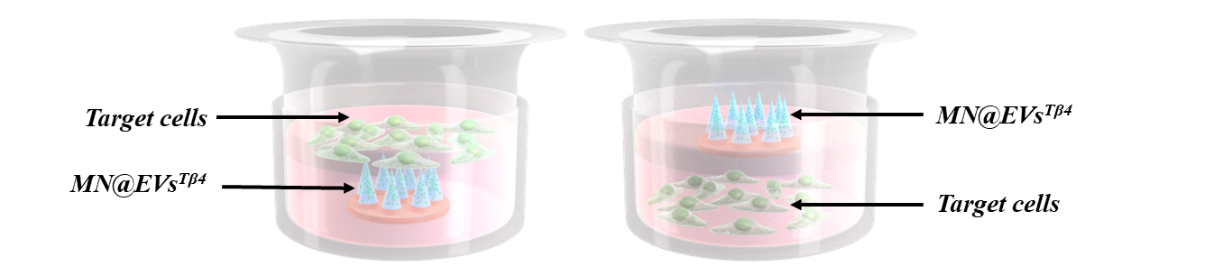


**Figure S6.** Schematic illustration of coculture of cells (HUVECs or HDFs) with MN@EVs^Tβ4^.


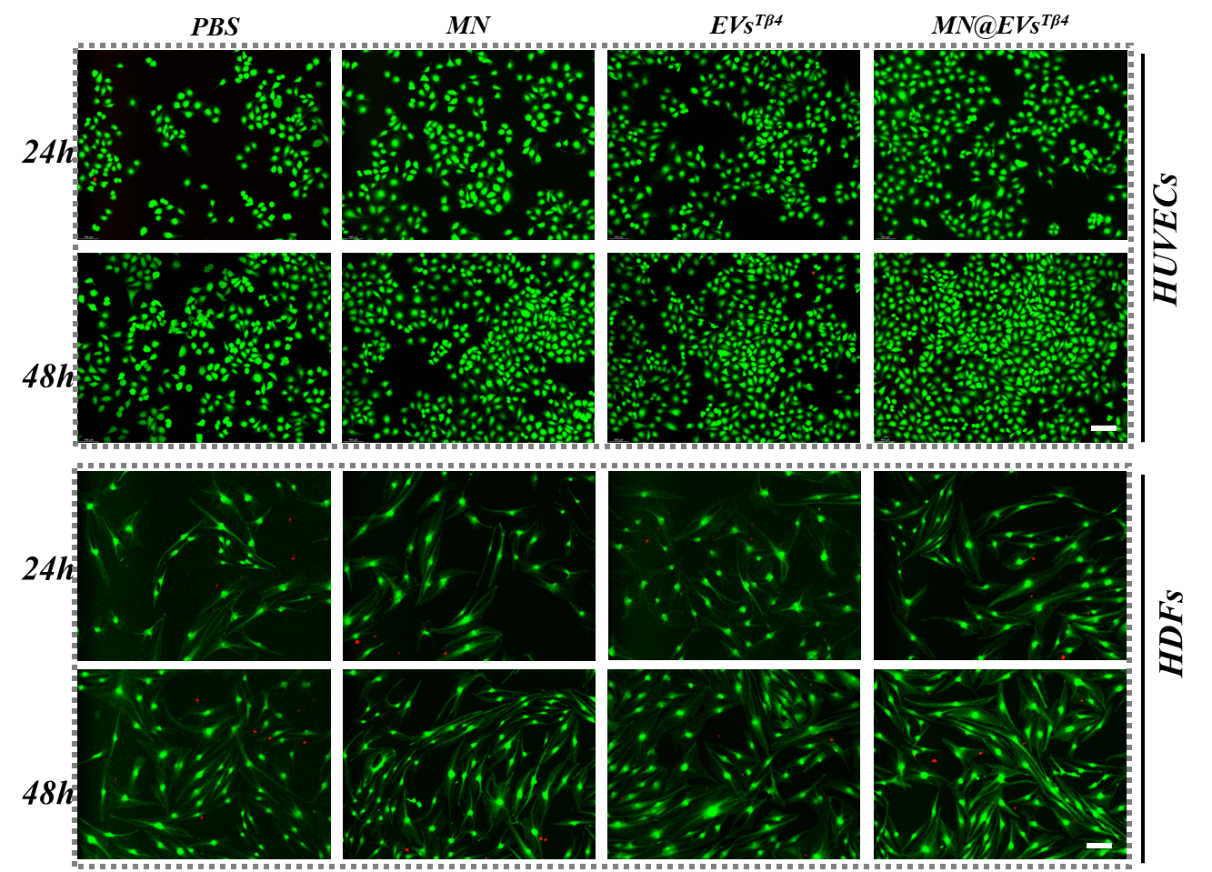


**Figure S7.** Live/dead cell staining images of HDFs, HUVECs cells co-cultured with

MN@EVs^Tβ4^. Scar bar = 100μm.


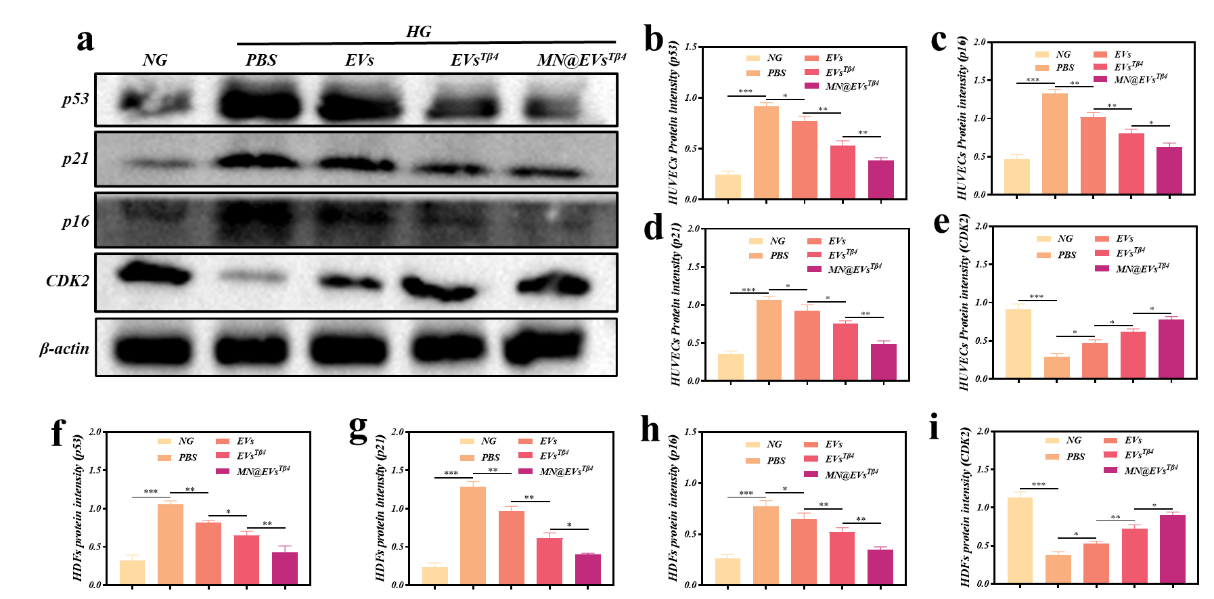


**Figure S8.** (a-e) Western blot (a) and quantification analysis (b-e) of the protein expression of p53, p21, p16 and CDK2 in HUVECs across different groups. n = 3. (f-i) Quantification of protein levels of p53 (f), p21 (g), p16 (h) and CDK2 (i) in HDFs in different groups. n = 3.


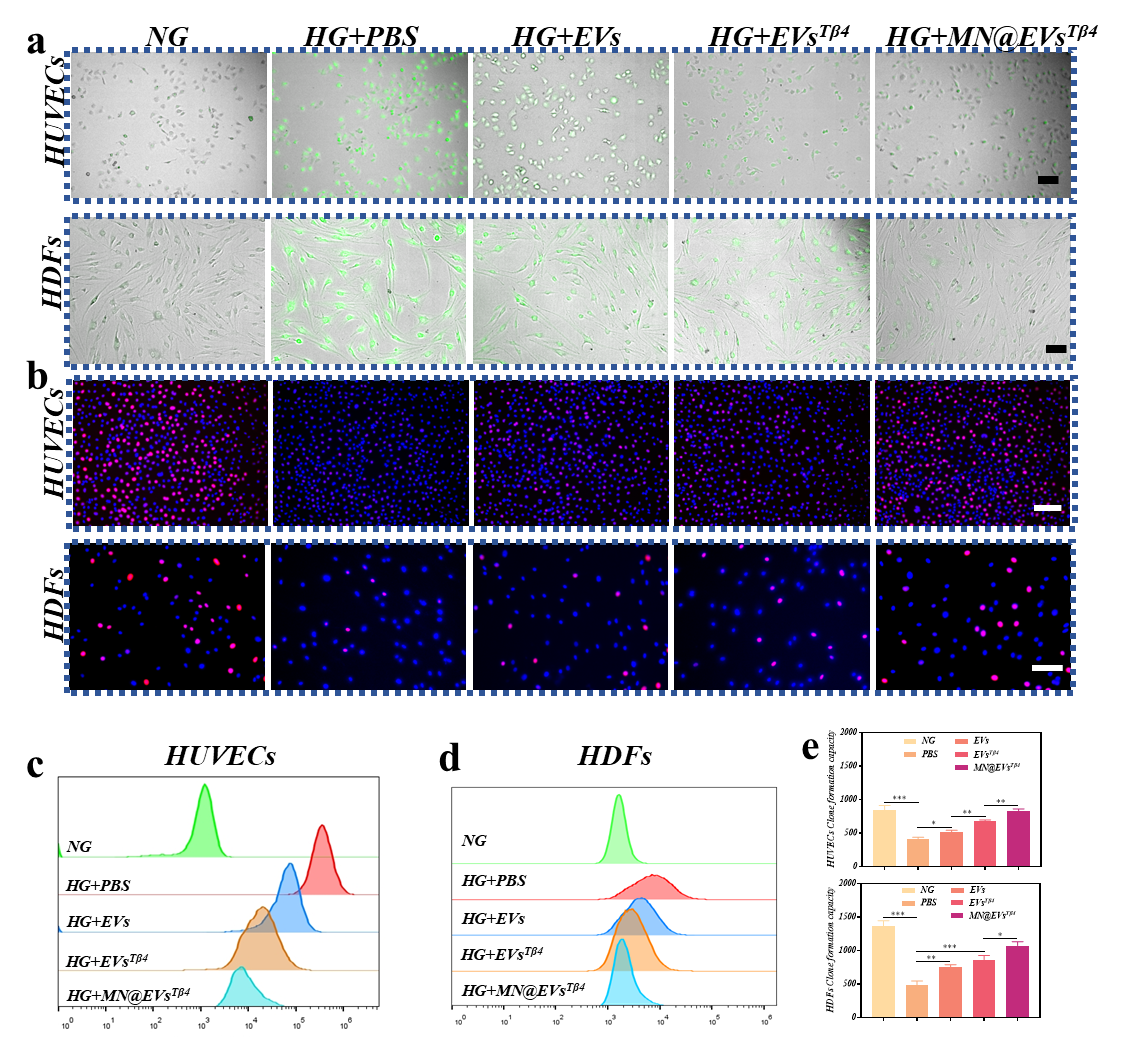


**Figure S9.** (a) ROS level treated with different groups estimated by DCFH-DA in HUVECs and HDFs. Scale bar = 100 μm. (b) The images of EdU staining across different groups. Scale bar = 100 μm. (c) ROS generation in HUVECs co-cultured with MN@EVs^Tβ4^ detected via flow cytometry using DCFH-DA. (d) ROS generation in HDFs co-cultured with MN@EVs^Tβ4^ detected via flow cytometry using DCFH-DA. (e) Quantification analysis of colony formation assays in HUVECs and HDFs in different groups. n = 3.


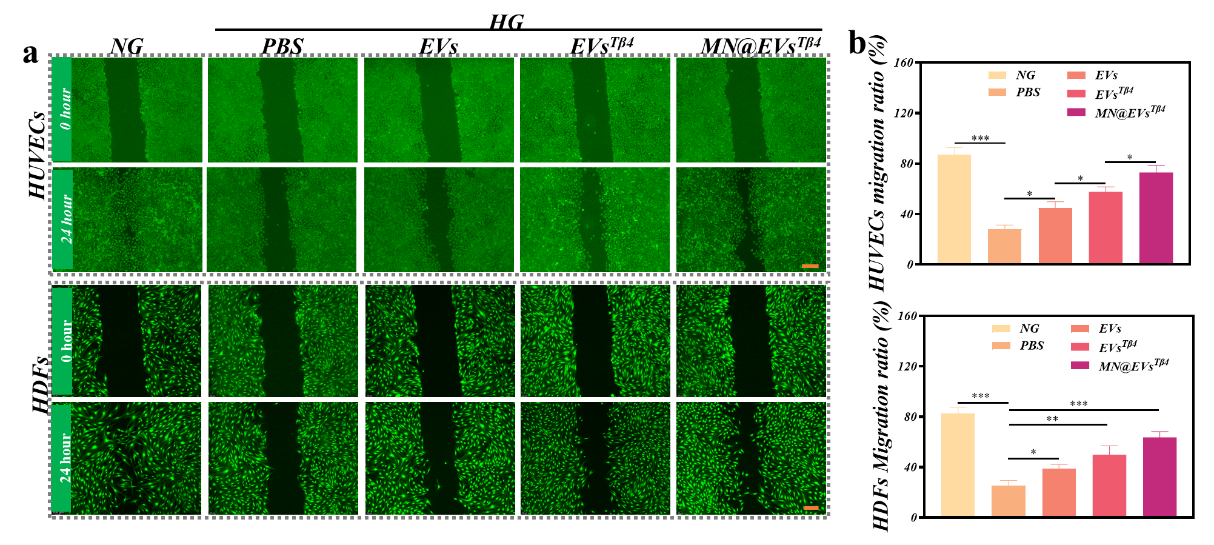


**Figure S10.** (a, b) The images (a) and quantification (b) of scratch assay reflecting migration abilities of HUVECs and HDFs with different treatments. n = 3, All scale bar = 100 µm.


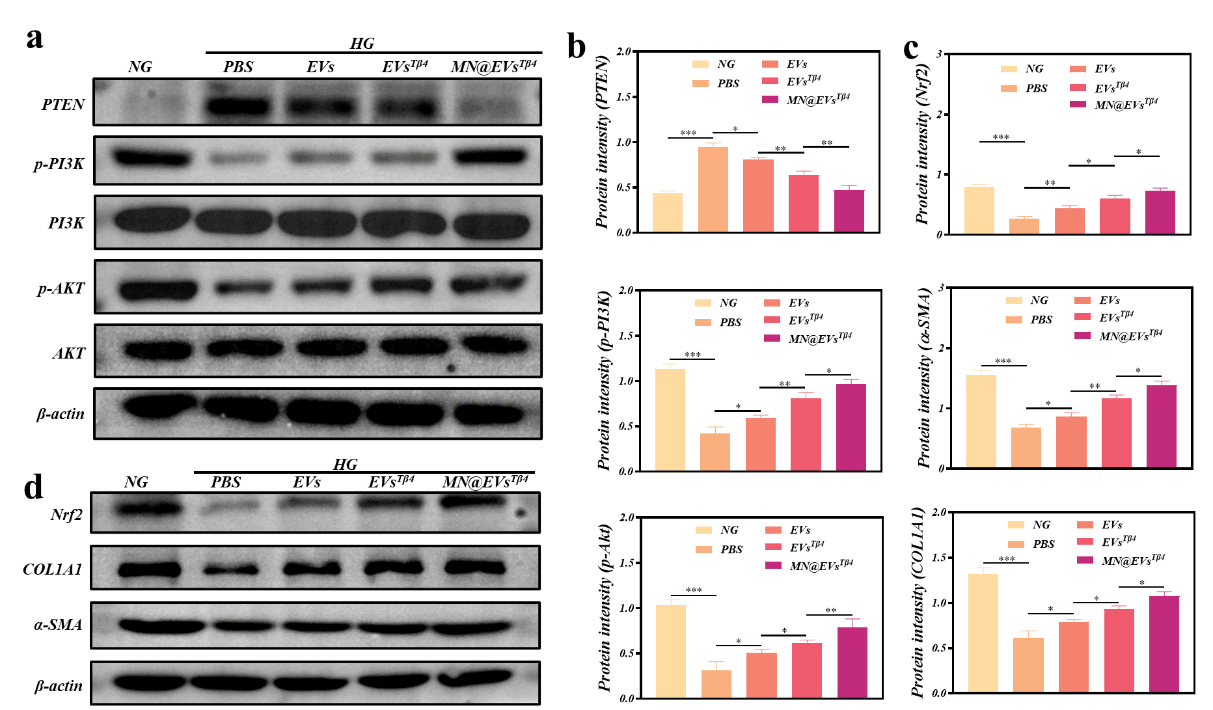


**Figure S11.** (a, b) Western blot and (a) quantification (b) of PTEN, p-PI3K, PI3K, p-AKT and AKT proteins expression in HDFs in different groups. n = 3. (c, d) Western blot (d) and quantification (c) of Nrf2, COL1A1 and α-SMA proteins expression in HDFs across different groups. n =3.


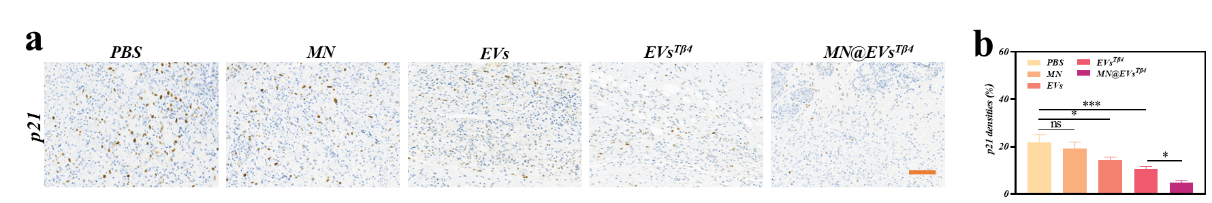


**Figure S12.** (a, b) Immunohistochemical staining (a) and quantification analysis (b) of p21 in different groups. Scale bar = 100 μm. n=3.


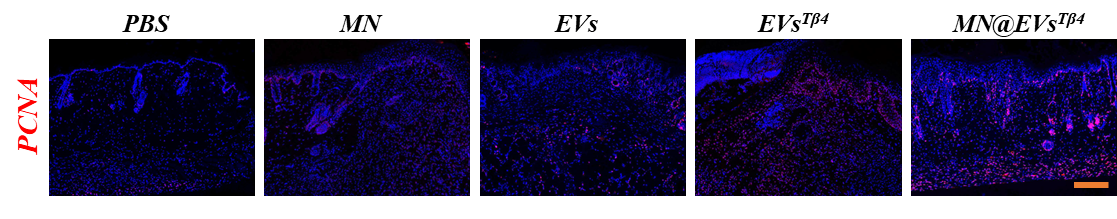


**Figure S13.** Immunofluorescence images of PCNA expression in tissues across different treatments. Scale bar = 100 μm.


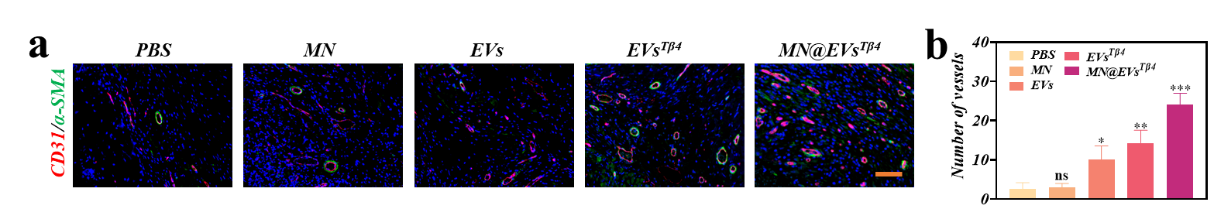


**Figure S14.** (a) Immunofluorescence images of CD31 (red) and α-SMA (green) expression and in tissues across different treatments. (b) Quantification of the number of vessels by immunofluorescent staining. Scale bar = 100 μm.


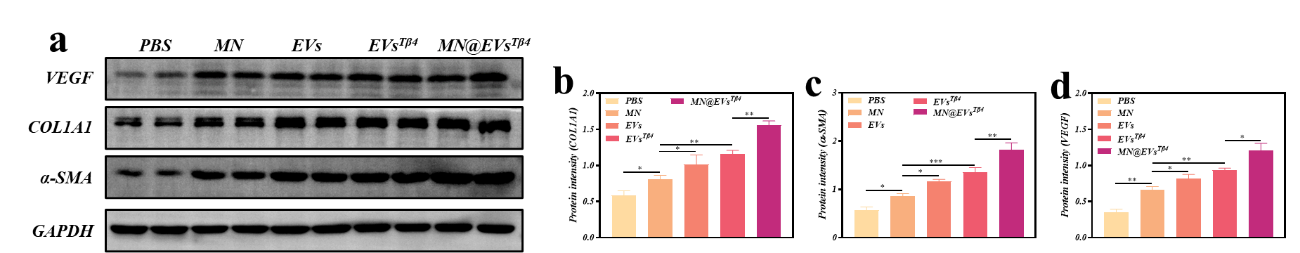


**Figure S15.** (a-d) Western blot (a) and quantification analysis (b-d) of protein levels of COL1A1, α-SMA and VEGF *in vivo* in different groups. n = 3.


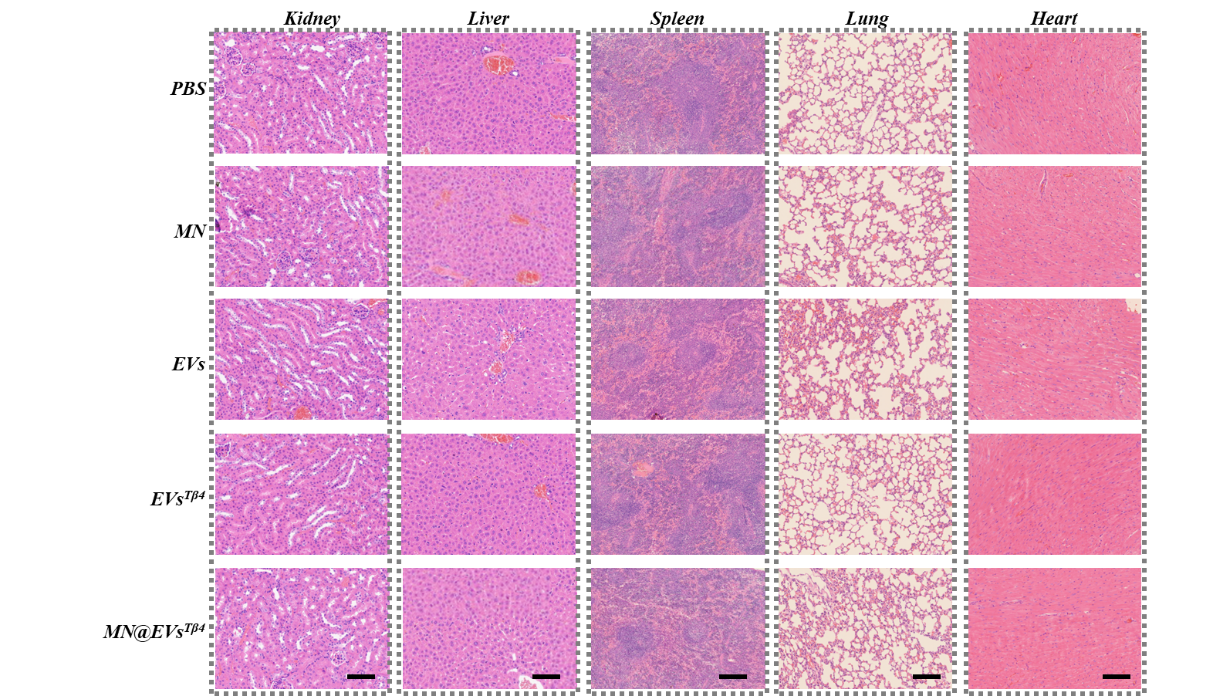


**Figure S16.** H&E staining results of major organs including kidney, liver, spleen, lung, and heart. All scale bars = 100 μm.

**Video S1.** Real-time recording of the entire process of MN@EVs^Tβ4^ tips detaching after the dissolution of the base layer
